# Fixed and Flexible: Coexistence of Obligate and Facultative Migratory Strategies in a Freshwater Fish

**DOI:** 10.1371/journal.pone.0090294

**Published:** 2014-03-03

**Authors:** Jakob Brodersen, Ben B. Chapman, P. Anders Nilsson, Christian Skov, Lars-Anders Hansson, Christer Brönmark

**Affiliations:** 1 Department of Biology, Lund University, Lund, Sweden; 2 Department of Fish Ecology and Evolution, EAWAG Swiss Federal Institute of Aquatic Science and Technology, Center for Ecology, Evolution and Biogeochemistry, Kastanienbaum, Switzerland; 3 National Institute of Aquatic Resources, Technical University of Denmark (DTU), Silkeborg, Denmark; Liverpool John Moores University, United Kingdom

## Abstract

Migration is an important event in many animal life histories, but the degree to which individual animals participate in seasonal migrations often varies within populations. The powerful ecological and evolutionary consequences of such partial migration are now well documented, but the underlying mechanisms are still heavily debated. One potential mechanism of partial migration is between-individual variation in body condition, where animals in poor condition cannot pay the costs of migration and hence adopt a resident strategy. However, underlying intrinsic traits may overrule such environmental influence, dictating individual consistency in migratory patterns. Unfortunately, field tests of individual consistency compared to the importance of individual condition on migratory propensity are rare. Here we analyse 6 years of field data on roach migration, gathered by tagging almost 3000 individual fish and monitoring their seasonal migrations over extended periods of time. Our aims were to provide a field test of the role of condition in wild fish for migratory decisions, and also to assess individual consistency in migratory tendency. Our analyses reveal that (1) migratory strategy, in terms of migration/residency, is highly consistent within individuals over time and (2) there is a positive relationship between condition and the probability of migration, but only in individuals that adopt a migratory strategy at some point during their lives. However, life-long residents do not differ in condition to migrants, hence body condition is only a good predictor of migratory tendency in fish with migratory phenotypes and not a more general determinant of migratory tendency for the population. As resident individuals can achieve very high body condition and still remain resident, we suggest that our data provides some of the first field evidence to show that both facultative and obligate strategies can co-exist within populations of migratory animals.

## Introduction

Migration is a central part in the life history of many animals [1]–[3], where migrants can benefit from temporal variation in the profitability of different habitats [4], [5]. However, knowledge is scarce regarding why only some individuals within populations migrate: a phenomenon known as partial migration [6], [7]. Partial migration is widespread among animals [7], [8], and can influence both trophic dynamics [9], [10] and population divergence [11], [12], suggesting that knowledge on the underlying mechanisms is much needed [7], [13].

A quarter of a century ago Lundberg [14] suggested that partial migration is controlled by a combination of fixed and variable factors. Since then, much focus has been on genetic versus environmental control, suggesting that partial migration should either be described as a facultative or an obligate behaviour [15]–[17]. However, more recent theory has suggested, as Lundberg did, that both environmental and genetic influences have to be taken into account when considering mechanisms of partial migration [18]. Irrespective of the relative importance of genetic and environmental factors, migration is generally considered to be caused by seasonal or ontogenetic changes in habitat-specific cost-benefit tradeoffs. Moreover, environmental variability may drive partial migration through relative success of residents and migrants dependent on their somatic condition [15].

Given a combined influence of genotype and environment on individual migratory decision, all individual organisms can be divided into three groups depending on their migratory strategy. These universal individual migratory strategies (UIMS) include obligate migrants (OM) that migrate irrespectively of environmental and individual conditions, facultative migrants (FM) that potentially migrate depending on environmental and individual conditions, and obligate residents (OR) that stay resident irrespective of environmental and individual conditions.

Like many other animal taxa [19], partial migration is often found in fishes [7]. Many freshwater fish, such as roach (*Rutilus rutilus* L.), show partial migration during winter, migrating from the lake system into connected streams, and returning to the lake the following spring [15], [20]. In contrast to many bird species performing partial migration, cyprinid fish are not considered territorial, and defending territories is therefore unlikely to influence partial migration in cyprinid populations, i.e. cyprinid fish do not remain resident to maintain a territory. Instead, the migration appears to be driven by a differential habitat-specific seasonal change in the relationship between predation risk and growth rate [5], where the streams and wetlands during winter offer a relatively safer, but poorer foraging habitat as compared to the lake [5], [20], [21].

Partial migration in roach, hence, appears to be an escape from predatory fish and avian predators [5], [20], where well-fed individuals with high somatic condition can afford the overwinter stay in the streams [15]. But individual proneness to migration cannot be explained entirely by somatic condition and differential access to food [15]. A significant amount of the variation between migrants and residents still remains unexplained, and recent data showing differences in individual risk-taking behavior between migrants and residents is suggestive of more fixed underlying differences between residents and migrants [22]. Within the group of migratory roach, we have shown consistency in migratory traits, such as their individual timing of migration and stream of destination, i.e. variables in differential migration [23], but whether there is individual consistency of having a migratory or resident strategy in the long term is still unexplored.

Whereas previous studies have often focused on the heritability of migratory propensity to show a genetic influence upon partial migration [24] or on experimental manipulation of the environment to show influence of environmental factors on partial migration [15], [25], we here take an observational approach, where we study natural variation in somatic condition and its effect on individual migration in combination with individual consistency in migration/residency. We analyse individual migratory data from almost 3000 fish from a partially migratory population over six consecutive years using a passive telemetry system. We test the hypothesis that somatic condition predicts migratory tendency in natural populations and also analyse our data to assess whether individuals are consistent between years in their migratory tendency.

## Materials And Methods

### Study system

The current study was conducted in Lake Krankesjön, a 3.4 km^2^, shallow (maximum depth 3.0 m) lake in southern Sweden, with a fish community dominated by a partially migratory roach population (for lake description, see [15]).

**Figure 1:**
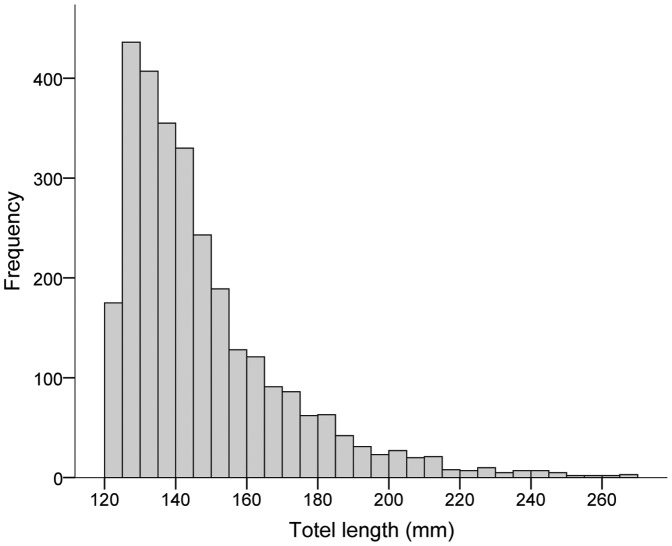
Size distribution of all tagged roach in Lake Krankesjön from 2003 to 2007. Note missing data for fish smaller than 120mm, which were too small to be tagged. As all captured individuals above 125mm were tagged, the data is representative for the general size distribution within the population and consistent with survey data from the lake.

Individual roach have been found to live up to 17 years in similar types of lakes in the proximity of Lake Krankesjön [26], but only very few individuals will achieve such a high age due to predation by a multitude of predators. The predators include piscivorous fish, such as pike (*Esox lucius* L.) and large perch (*Perca fluviatilis* L.) [5] and piscivorous birds, such as cormorants (*Phalocrocorax carbo* L.) [20]. In Lake Krankesjön, the roach population is dominated by relatively small individuals (<200 mm TL; Fig. 1). Roach are generally considered omnivorous and are known to feed on zooplankton, benthic invertebrates, detritus and plants [21]. During the migration period, resident individuals generally feed on higher quality food items than migrants [21].

### Fish tagging and monitoring of migration

Roach were caught by electrofishing shortly before the migration periods started between September 22^nd^ and November 23^rd^ each year from 2003 through 2007. We restricted tagging to this period to reduce the effect of mortality before migration. The number of fish tagged each year varied between 480 and 696 and in total we have tagged 2909 individuals. Each year fish were caught over the whole lake in open water, submerged vegetation, and littoral habitats with the majority caught at night in open water over submerged vegetation. However, the different habitats are in close proximity and fish are known to regularly change habitats, e.g. during the diel cycle [27]. Further, we have not observed any difference in migration patterns of fish caught in different habitats (J. Brodersen, unpublished analyses). Hence, although we have only tagged a subset of the fish in the lake, we consider them to be representative of the whole population.

After capture, fish were stored in net enclosures overnight and tagged on the following day. After being weighed to nearest 0.1 g and measured to nearest mm for total length (mean±SD: 147 mm±23.3; range: 120–268 mm), all fish were tagged according to Skov et al. [28] by surgically implanting a TIRIS Passive Integrated Transponder-tag (PIT-tag) (Texas Instruments, RI-TRP-RRHP, half duplex, 134 kHz, 23.1 mm long, 3.85 mm diameter, 0.6 g in air) into the stomach cavity of the fish. After tagging, all fish were released into the lake at the approximate area of capture.

Directional migration of fish between the lake and the inlet and outlet streams was monitored with a modified PIT-tag antenna system consisting of two antennas and a recording station in each stream (for details see [15]). Migration was monitored from October 2003 until June 2009, i.e. for six consecutive migration periods. Due to the nature of the nature of the telemetry system, i.e. passive telemetry, only fish that migrated were registered on the antennas. It is therefore not possible to determine whether fish that do not migrates are at a given time resident in the lake or dead. The migration data therefore has to be treated with care before interpreting individual differences in migration/residency patterns (see *Data treatment* section below).

An evaluation of PIT-tag marking techniques showed that this method results in no detrimental effects to fish, including no adverse impact upon body condition [28]. The study complies with the current laws in Sweden; ethical concerns on care and use of experimental animals were followed under permissions (M14-04 and M165-07) from the Malmö/Lund Ethical Committee. Permissions for carrying out fieldwork at Lake Krankesjön were granted by the Revingehed military command (P7). No endangered species or species of concern was used in the study.

### Data treatment

Some of the differences in migratory propensity in later years between fish that either migrated or stayed resident during the first year after tagging can be attributed to different over-winter mortality in the lake and in the streams, with over-winter mortality expected to be higher in the lake as compared to the stream [20]. Direct comparisons of continued participation in migration over longer time periods, i.e. several years, between first year migrants and residents may therefore be biased towards a higher continued participation in migration by fish that migrated during their first year after tagging. A more accurate approach to test for individual consistency in migratory propensity is to evaluate if individuals that migrated the second winter after tagging also migrated the first, and compare this ratio of first-year migrants with the ratio of first-year migrants for the whole population using a χ^2^-test. We will refer to this approach as the mortality corrected approach.

For analysis of migration/residency patterns over multiple years, we classified individual fish as migrants if they migrated in either the first or the second winter after tagging, since data on migration in the third to sixth winter after tagging were only available for fish tagged during the first year of the study (2003). This procedure is justified by the observation that only very few fish (0.2%) that did not migrate in either the first or the second winter after tagging would engage in migration later in their life. Dividing fish into migratory groups depending on whether they participated in migration in the first and the second year after tagging results in four different groups: NN, NY, YN and YY, with letters indicating individual migration (Yes or No), in first and second winter after tagging respectively (Table 1). For interpretation of the potential influence of environment, genotype and mortality on observed migration patterns in the different groups, see Table 1.

**Table 1:** Grouping of individuals based on observed migration in first (Y1)- and second year (Y2) after tagging and associated potential universal individual migration strategies (UIMS) and survival.

Group	Migration Y1	Migration Y2	UIMS potentially in group	Potential dead or alive	*N*
YY	Yes	Yes	OM & FM	Alive both years	464
YN	Yes	No	OM & FM	Alive Y1. Potentially dead Y2	1223
NY	No	Yes	FM	Alive both years	70
NN	No	No	OM, FM & OR	Potentially dead both years.	1152

The three UIMS include obligate migrantion (OM), facultative migrantion (FM) and obligate residency (OR). See text for further explanation. *N* refers to the number of individuals in the different groups in the present study.

For analyses of the effects of individual condition on partial migration we used residual values from a linear regression between fish total length and Fulton's *K* (*K* = 100000 *m* / TL^3^; where *m* is body mass in gram and TL is fish total length in mm), to control for a general increase in Fulton *K* with fish size [15]. This measure of relative condition will henceforth be referred to as somatic condition.

Since measurements of individual somatic condition were only possible during the individual tagging event, we only view somatic condition as a reliable predictor for migration in the first winter after tagging. In subsequent years, individual somatic condition is likely to have changed and somatic condition at tagging would therefore not be expected to directly influence migration in subsequent years. However, if some individuals are obligate residents, then these fish would not be expected to migrate even when in high somatic condition (see Table 1). This would lead to the expectation that the resident fish would consist of facultative migrants in low condition and obligate residents that on average are in a relatively higher condition. Hence, we would expect a conditional difference between first year resident fish that would later in their life initiate migration (group NY, consisting entirely of facultative migrants (Table 1)) and first year resident fish that would never initiate migration (group NN, consisting potentially of a mix of facultative migrants and obligate residents (Table 1)). Alternatively, if resident fish consisted exclusively of facultative migrants, we would not expect a conditional difference between these groups.

We analysed the effect of somatic condition on individual participation in migration during the first winter only in individuals that were found to migrate in the second winter after tagging (NY and YY) using binary logistic regression, with likelihood ratio backward selection of the variables somatic condition and fish length (selection criteria *p*<0.1). We here corrected for potential ontogenetic effects by including individual fish length as a covariate in the analysis. However, since there was no significant (*p* = 0.698) effect of length, it was not included in subsequent analyses. Using only these two groups (NY and YY) rules out any unwanted effects of mortality during the first year after tagging and also focuses only on fish that are genetically predisposed for migration, without genotype necessarily dictates migration. We subsequently test for differences in somatic condition between all groups using one-way ANOVA with post hoc tests of between-group differences.

## Results

### Individual consistency in migratory propensity

Fish migrating the second year after tagging were significantly more likely to have migrated in the previous year as compared to the whole population (χ^2^ = 160.7; *p*<0.001). Specifically, 86.9% of the second year migrants had migrated during the first winter after tagging, whereas this was only the case for 58.0% of all fish together.

When backtracking migration history of fish that migrated several years after tagging the differences become even more evident (Table 2). In total over all years, only 13.1% out of the 534 tagged fish that migrated during the second winter after tagging did not migrate during the first winter. Further, of 121 fish that migrated both in the first and in the third winter after tagging, only one individual did not migrate in the second winter after tagging, i.e. switched from migration to residency and back again. We therefore conclude that individual consistency is higher than predicted from random assignment of migration.

**Table 2:** Migration frequencies during preceding years (columns) of fish found to migrate at winters several years after tagging (rows).

Winter after tagging	5^th^	4^th^	3^rd^	2^nd^	1^st^
6^th^ (*N* = 4)	100%	100%	100%	100%	75%
5^th^ (*N* = 10)		100%	100%	90%	80%
4^th^ (*N* = 43)			100%	**97.7%**	69.8%
3^rd^ (*N* = 155)				96.1%	78.1%
2^nd^ (*N* = 534)					86.9%

For example, out of the 43 tagged fish found to migrate in the fourth winter after tagging (aggregated for several tagging years) 97.7% were found also to have migrated in their second winter after tagging (bolded).

### Is migration condition-dependent?

**Figure 2:**
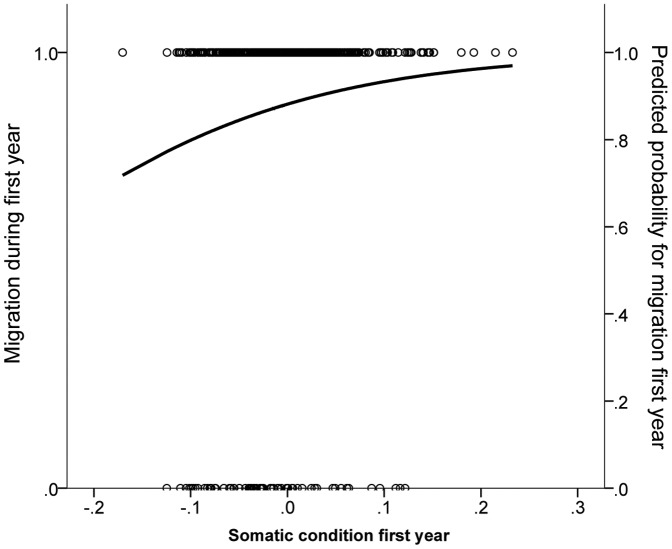
The effect of somatic condition on the probability of migration in the first winter after tagging for roach migrating at least once in subsequent winters. A higher somatic condition at the time of tagging increases the likelihood of migration during the first winter after tagging. Circles indicate observed individual participation in migration during the first year after tagging (1: Migration; 0: Residency), whereas line indicate predicted probability of migration based on observed values.

At the population level, contrary to our initial prediction, we found no effect of somatic condition on the probability to migrate during the first winter after tagging (logistic regression; Wald  = 0.031; *p* = 0.86). However, we found a clear positive relationship between somatic condition and migration during the first winter after tagging in all individuals that migrated during the second winter after tagging (groups NY and YY) (logistic regression; Wald  = 6.47; *p* = 0.011; Fig. 2). Hence, body condition is important in migratory decision-making for individuals with a potential migratory strategy; potential migrants in better condition are more likely to migrate than those in poor condition.

**Figure 3:**
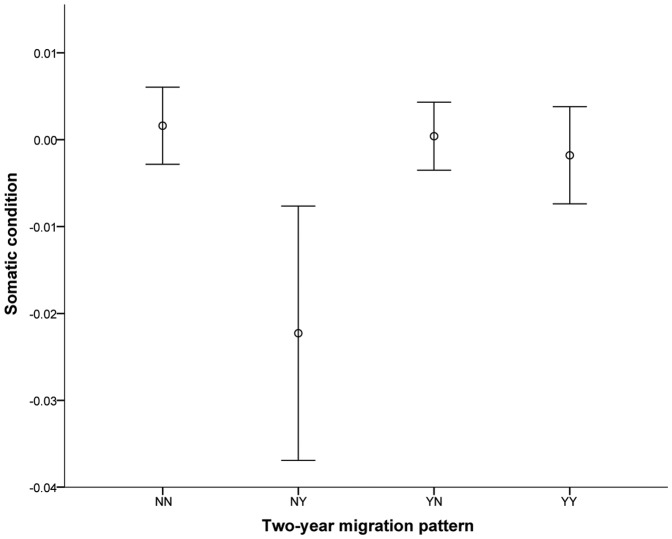
Mean condition of four groups of individuals based on their residency (N) or migration (Y) during each of the first two years after tagging. Error bars indicate 95% confidence interval of the mean. Fish that changed from residency to migration (NY) were in significantly lower condition as compared to other groups.

When dividing individuals into four groups based on whether they migrated during the first and second winters after tagging, we found a strong tendency for groups being in different somatic condition (ANOVA; *F* = 2.50; *p* = 0.058; Fig. 3). Subsequent post hoc tests revealed that individuals that changed from residency to migration (group NY) have significantly lower somatic condition in the first year than fish that would never participate in migration later in their life (group NN) (Tukey HSD; *p* = 0.038; Table 3). In fact, fish that would never migrated had on average a higher somatic condition than any of the other groups, although only significantly different from the NY group (Table 3).

**Table 3:** Post hoc (tukey HSD) table for ANOVA test of between group difference in somatic condition.

(I) Group	(J) Group	Mean Difference (I-J)	Std. Error	Sig.
NN	NY	.024*	0.0089	0.038
	YN	0.001	0.0029	0.976
	YY	0.003	0.0039	0.822
NY	YN	−0.023	0.0089	0.054
	YY	−0.02	0.0093	0.123
YN	YY	0.002	0.0039	0.942

Groups correspond to migration patterns during the first two winters of tagged fish: Yes (Y) and No (N). For further explanation of groups see text and Table 1. Redundant comparisons are removed from the table.

*The mean difference is significant at the 0.05 level.

## Discussion

By following a high number of individually tagged fish over multiple years we found that individual fish show high individual consistency in their migratory behaviour in terms of a migratory or resident strategy. Such individual consistency can be caused by either underlying genetically determined differences in proneness to migration or due to initial plasticity followed by canalization into a migratory or resident phenotype [19]. This canalization can potentially occur if individual over-wintering success is evaluated based on previous individual experiences. In such a scenario, where individual success criterion is survival, individuals are expected to be strongly biased towards consistency, since only surviving individuals get to make a second choice. Therefore, based on individual consistency alone, we cannot infer whether the underlying mechanism is due to canalization or underlying genetic differences. However, combining the consistency results with data on individual somatic condition brings us a step closer to such conclusions.

We found clear but complex effects of condition on the individual participation in the seasonal migration. By analyzing only fish that were known to migrate in the second year after tagging, i.e. fish that we could be sure of would be both predisposed for migration and were also alive the first winter after tagging, we found that somatic condition was positively related to the propensity to migrate in the first year after tagging. We interpret this as a pre-migratory decision based on somatic condition among fish that have a migratory phenotype, where fish in poor somatic condition will be more likely to choose to stay in the lake as compared to fish with high somatic condition. This fits well with an earlier experimental study showing that experimentally fed fish are more likely to migrate than unfed fish [15]. However, the same study showed that despite this strong positive influence of feeding on migratory propensity, a high proportion of unfed fish also migrated and, further, about 10–15% of fed fish did not [15]. This latter observation from experimental studies corroborates our current result, that long-time resident fish are not in lower condition than migratory fish (see below).

The differences in likelihood of migration between second year migrants and the whole population may be argued to be due to mortality in the pre-migration period. However, this would mean that almost 30% of the whole population would die in the period between tagging and migration. We see this as highly unlikely, since this period is relatively short (median tagging date: October 5; median outmigration date: November 15) and since most of the predation is expected to occur in the months prior to tagging, when the water temperature is higher [5]. Furthermore, one of the groups (NN) is influenced by mortality during the first year after tagging. Since mortality is generally higher in fish with lower somatic condition, e.g. due to starvation or increased risk taking leading to higher predation risk [29], [30], we would expect that mortality effects would only potentially cause the analysis to show higher somatic condition in first year residents that would migrate during the second year after tagging (group NY) as compared to the first year residents that would also not migrate in subsequent years (group NN). Also somatic conditional carry-over effects would give this outcome of the analysis. However, a higher somatic condition in the NN-group as compared to the NY group, as found in the current study, can be caused by fish that are not predisposed to migration, where somatic condition does not influence individual participation in migration, i.e. obligate residents.

Our results suggest that energetic constraints are important for fish that adopt a migratory phenotype, but that condition per se does not explain patterns of partial migration in this system. This is due to the, on average, relatively high somatic condition of life-long resident fish and we interpret this as coexistence of fixed (here residency) and flexible (migration/residency) strategies within a single population. This further raises the question on whether resident and migratory fish differ in other traits, such as habitat occupancy, anti-predator behaviour and foraging niche. To our knowledge, all roach spawn sympatrically in the lake (K. Hulthén & B.B. Chapman, in prep), but since selection may favor different traits in the lake and in the stream, it appears likely that individuals with e.g. morphological traits corresponding to their migratory phenotype would have an advantage towards individuals, with no link between migratory phenotype and morphological traits related to different fitness in the different overwintering habitats. At this point, we know that migrants and residents differ in their underlying behavioural types [20], but further differences are currently unknown; ongoing work tries to reveal differences between residents and migrants when they coexist in the lake during summer.

Our data supports Lundberg's more nuanced view of partial migration [14], suggesting coexistence of both fixed (in the current study for residency) and flexible (migration/residency) strategies within a population. Irrespective of somatic condition, some fish never migrate, and hence have a fixed resident strategy, whereas individuals with a migratory phenotype migrate with a higher probability when they are in better condition. To our knowledge this study provides one of the first examples of field data in support of Lundberg's [14] suggestion. Recent conceptual work also suggests that migration can be both fixed and flexible, and has attempted to reconcile these putatively opposing types of partial migration (Environmental Threshold Model [18]). In this model, obligate residents and migrants are individuals with a liability, respectively, much below and above an environmentally induced migration threshold point, whereas facultative migrants are individuals with liabilities close to the threshold. For such facultative individuals, migration is believed to depend on environmental factors [18], in accordance with our results. Contrasting to this, individuals with an obligate migration strategy, i.e. either fixed residents or fixed migrants, will follow their fixed strategy irrespective of environmental factors. Whereas our data suggest the existence of obligate residents within the studied roach population, we can with our data not test whether obligate migrants exist within the population. From the relatively high migration of starved fish in previous studies [15], it does, however, appear very likely.

We conclude that in at least partially migrating roach, some individuals may never migrate, irrespective of somatic condition, suggesting a fixed resident strategy, whereas migratory phenotypes migrate with a higher probability when they are in better condition. We suggest that similar patterns of co-existing fixed and flexible strategies may be found in other species as well.
